# Cervical Spine Pseudogout Mimicking Ossification of the Posterior Longitudinal Ligament: A Case Report and Literature Review

**DOI:** 10.7759/cureus.79790

**Published:** 2025-02-27

**Authors:** Shao-Lun Chen, Ping-Chuan Liu, Wen-Cheng Huang

**Affiliations:** 1 Department of Neurosurgery, Taipei Veterans General Hospital, Taipei, TWN

**Keywords:** calcium pyrophosphate deposition disease (cppd), cervical-spine, compressive myelopathy, ligamentum flavum calcification, ossification of the posterior longitudinal ligament (opll)

## Abstract

Calcium pyrophosphate deposition (CPPD) disease, commonly known as pseudogout, is characterized by calcium pyrophosphate crystal accumulation in joint tissues. While CPPD typically affects peripheral joints, cervical spine involvement is rare but significant due to potential spinal cord and nerve root compression, leading to myelopathy and radiculopathy. Here, we describe a 69-year-old female who presented with progressive cervical myelopathy and extensive calcifications in the cervical ligamentum flavum (CLF), posterior longitudinal ligament (PLL), and intervertebral discs. These findings initially resembled ossification of the posterior longitudinal ligament (OPLL), a much more prevalent pathology. Surgical decompression through both anterior and posterior approaches relieved the compression. Pathological evaluation confirmed CPPD via the identification of weakly positive birefringent crystals. Postoperatively, the patient’s symptoms improved significantly, although persistent right shoulder pain suggested CPPD in peripheral joints. This report underscores the rarity of cervical CPPD with widespread ligament and disc involvement, emphasizing that it can mimic common degenerative diseases like OPLL. Early recognition and surgical intervention are imperative for preventing irreversible spinal cord damage and achieving favorable outcomes. In addition, the persistent right shoulder pain illustrates that CPPD can occur at multiple sites and requires a holistic approach to patient care.

## Introduction

Calcium pyrophosphate deposition (CPPD) disease is characterized by the accumulation of calcium pyrophosphate crystals within affected tissues, which display weakly positive birefringence and rhomboidal shapes under polarized microscopy [1]. When these crystals provoke an inflammatory response, the condition is termed pseudogout. Although CPPD primarily affects peripheral joints like the knees and shoulders, its involvement in the cervical spine is rare but clinically meaningful [2]. The likelihood of developing CPPD increases with age and is predominantly observed in individuals over 65 years [3]. Although CPPD often appears idiopathic, metabolic conditions such as hyperparathyroidism, hemochromatosis, hypothyroidism, and hypomagnesemia, as well as certain genetic predispositions, can elevate this risk [4].

Cervical spine CPPD can present in various ways, from asymptomatic calcifications to acute inflammatory episodes or chronic degeneration that leads to spinal cord compression [3,5]. Notably, calcium deposits in the cervical spine have been observed across different structures, with the transverse ligament of the atlas and the cervical ligamentum flavum (CLF) being commonly affected [5-7]. Clinical symptoms of cervical spine CPPD often overlap with those of more prevalent inflammatory or degenerative disorders, complicating diagnosis and underscoring the importance of differential diagnosis.

This case report describes a rare presentation of cervical CPPD involving the CLF, posterior longitudinal ligament (PLL), and intervertebral discs, culminating in cervical myelopathy. This case emphasizes the importance of recognizing CPPD as a potential cause of cervical myelopathy and demonstrates the effectiveness of surgical decompression for neurological recovery.

## Case presentation

A 69-year-old woman presented with a two-year history of progressive neck pain radiating to the right arm, numbness in her hands, and unsteady gait. She has a medical history of diabetes mellitus, hypertension, arrhythmia, and hyperthyroidism. Her symptoms had worsened in months, with increased gait imbalance and frequent falls. Physical examination revealed hyperreflexia in the upper limbs, positive Hoffman signs, and grip strength of 4/5 bilaterally, with normal muscle strength in other regions. Motor-evoked potential (MEP) studies suggested a bilateral corticospinal lesion above the C8 level.

**Figure 1:**
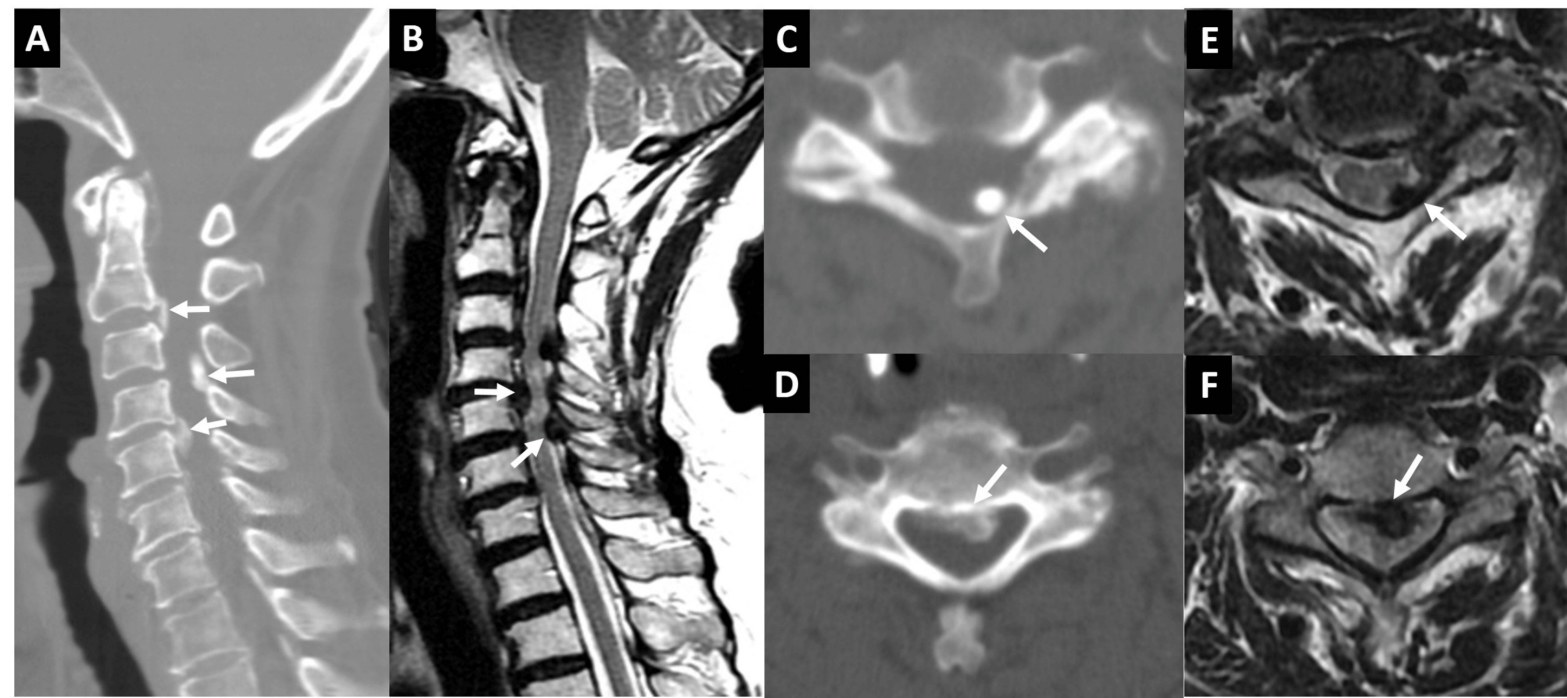
CT and MRI images of the cervical spine (A) A sagittal CT image shows protruding discs and calcified CLF, indicated by white arrows. There is a small calcific deposit in the atlantoaxial joint region. (B) A sagittal T2-weighted MRI reveals hypointense lesions arising from the discs, PLL, and CLF. An abnormally high signal intensity in the spinal cord implies myelopathy. White arrows indicate the protruding disc and CLF. (C, D) Axial CT images at C3-C4 (C) and C4-C5 (D) reveal narrowing of the spinal canal caused by the calcified CLF and calcified protruding disc, respectively, marked by white arrows. (E, F) Axial T2-weighted MRI at C3-C4 (E) and C4-C5 (F) reveal hypointense lesions arising from the discs and CLF. These lesions compress the spinal cord from anterior and posterior aspects, respectively, indicated by white arrows. CLF: cervical ligamentum flavum; PLL: posterior longitudinal ligament

Cervical spine CT revealed herniation of the nucleus pulposus at C2-C3 (Figure 1A), a protruded disc with CLF hypertrophy at C3-C4 (Figure 1C), and significant spinal stenosis. Additionally, herniation of the nucleus pulposus and calcification of the PLL were identified at C4-C5 (Figure 1D), further contributing to marked spinal stenosis. A cervical spine MRI demonstrated extensive spinal stenosis at C2-C6 (Figure 1B). At the C3-C4 level, the calcification of CLF compressed the spinal cord from the left posterior aspect (Figure 1E). Calcificated PLL and herniation of the nucleus pulposus impinged the spinal cord anteriorly (Figure 1F). A hyperintense T2 signal at the C4-C5 level indicated compressive myelopathy.

**Figure 2:**
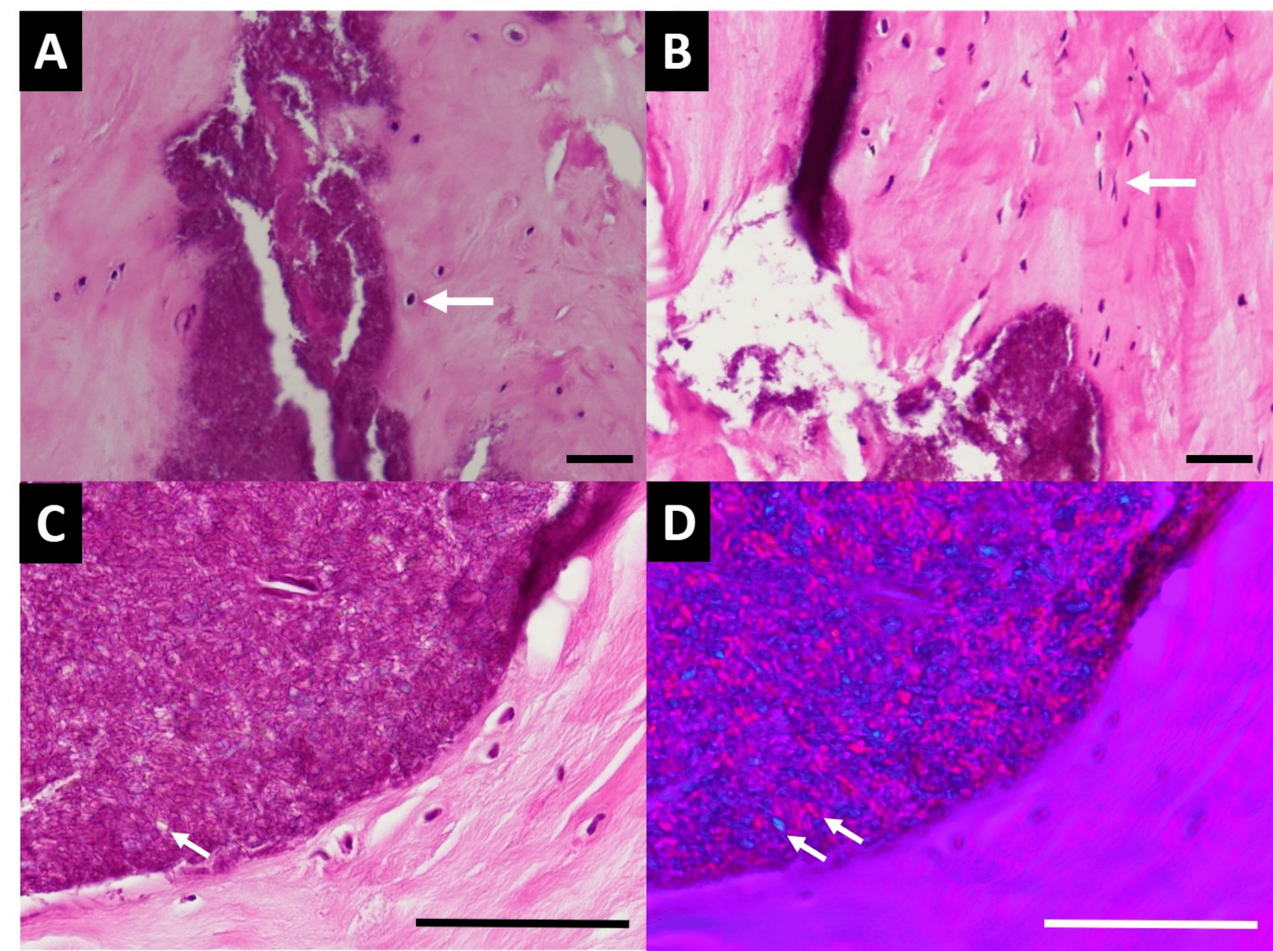
H&E-stained sections show CPPD in the cervical tissue (A) Small, round, or oval-shaped cells with centrally located nuclei are chondrocytes of the cervical disc, indicated by white arrows. Purple crystal deposits represent pseudogout material. (B) Purple crystal deposits are also found beside spindle cells of the CLF, marked by white arrows. (C) Crystal deposits exhibit a rhomboidal shape, indicated by white arrows. (D) Under polarized light, crystals display positive birefringence, marked by white arrows. (A-C) H&E staining; (D) polarized light microscopy; scale bar: 50 μm (A-D). CPPD: Calcium pyrophosphate deposition; CLF: cervical ligamentum flavum; H&E: hematoxylin and eosin

The patient underwent multilevel cervical decompression surgery, including C3-C6 discectomy, C4-C5 corpectomy, interbody fusion with cage and plate, and total laminectomy from C2 to C7 with posterolateral fusion over C3-C6. At the C4/C5 level, we observed that the calcified segment of the PLL displayed almost no clear tissue plane between itself and the dura. We used microdissection under an operating microscope to carefully peel the ossified or calcified tissue away from the dural surface in incremental steps. An ultrasonic bone scalpel was used to avoid mechanical vibration damage to the underlying spinal cord. The disc and PLL specimens were sent for pathological analysis. The disc and PLL specimens appeared grayish-white with a firm to hard texture. The CLF exhibited white-yellowish soft to firm tissue. The specimens were processed with hematoxylin and eosin (HE) staining. Histological examination revealed fibrocartilage with features of fibrillary degeneration, clumping of chondrocytes, and focal calcifications. Notably, calcium pyrophosphate crystal deposition, consistent with pseudogout, was identified within the fibrocartilage (Figure 2A), and also surrounding the ligament (Figure 2B). The crystals were characterized by purple aggregates of small rhomboidal crystals, which displayed positive birefringence under polarized light (Figures 2C, 2D).

**Figure 3:**
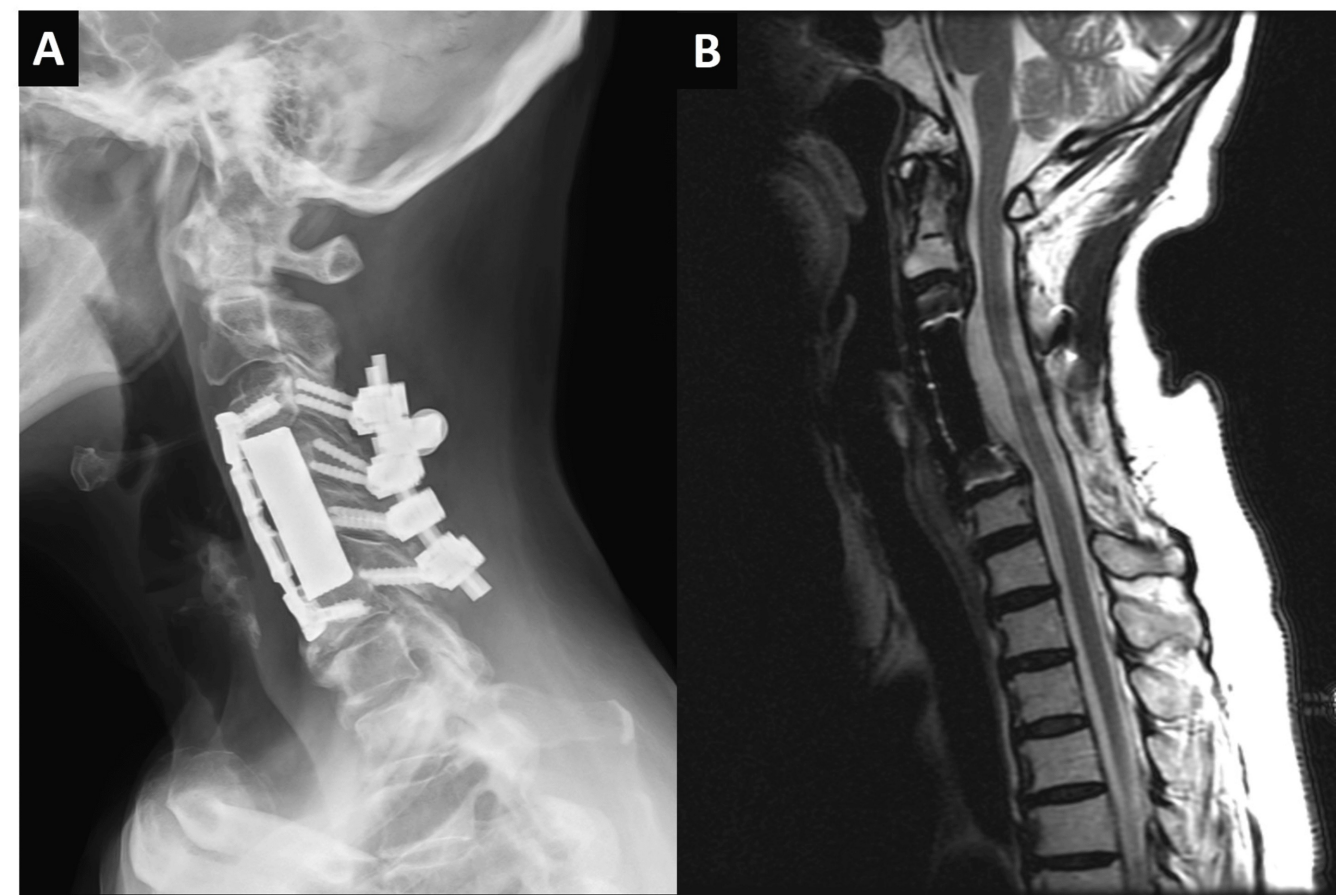
Postoperative follow-up at two years (A) A cervical spine X-ray shows adequate position and alignment of the fusion instruments. (B) A T2-weighted magnetic resonance image reveals satisfactory decompression of the spinal cord without recurrence of stenosis.

Postoperatively, her numbness and gait instability improved significantly, and she was discharged one week later. However, right shoulder pain persisted, and follow-up sonography identified a hyperechoic area on the supraspinatus tendon. This finding suggested possible CPPD in the shoulder joint. The pain was managed with nonsteroidal anti-inflammatory drugs (NSAIDs) and rehabilitation, and she remained stable at a two-year follow-up with no evidence of recurrence (Figure 3).

## Discussion

The cervical spine is an area of high mobility, with the CLF and PLL being especially elastic in this region. Over time, the thickness of these ligaments increases due to accumulated mechanical stress [8]. Because these ligaments are in direct contact with the dura mater, any abnormal hypertrophy has the potential to compress the spinal cord directly [9]. In cases of cervical spine CPPD, progressive joint degeneration and chondrocalcinosis can further exacerbate compression. Aggregated chondrocalcinosis deposits in the cervical spine may lead to compression of the spinal cord or nerve roots, manifesting as symptoms of myelopathy and radiculopathy [10].

Patients with cervical CPPD often experience neck pain, stiffness, limb numbness, and weakness. In acute cases, CPPD may present with fever and headache, potentially mimicking conditions such as meningitis [11]. Studies have shown that patients with CPPD are at a significantly higher risk of experiencing neck pain compared to the general population [3]. Imaging is essential for accurate diagnosis, with cervical CT highly sensitive for detecting calcific deposits and chondrocalcinosis [12]. Meanwhile, MRI is optimal for assessing soft tissue changes and neural compression in patients with cervical myelopathy symptoms [13]. CPPD nodules typically present as areas of low signal intensity on T1- and T2-weighted images, surrounded by high-intensity and medium-intensity signals indicative of edematous changes [14].

Within the cervical spine, CPPD most frequently affects the atlantoaxial joint, resulting in crowned dens syndrome, characterized by calcification around the odontoid process. According to previous studies, the overall prevalence of atlantoaxial CPPD crystal deposition may account for up to 12.5% of patients who have undergone a cervical spine CT scan, and the prevalence increases with age [6,7,11]. In our case, a small calcific deposit was noted in the atlantoaxial joint region, but it did not cause a significant mass effect or spinal cord compression. Hence, it was not the primary culprit for her symptoms, although it underscored the multifocal nature of the disease. Beyond the atlantoaxial region, calcifications can also develop elsewhere, particularly in the CLF, which accounts for approximately 95% of cases involving spinal cord compression [15]. Nevertheless, our current case stands out because the CPPD was observed in an unusual distribution, affecting not only the CLF, but also the PLL and intervertebral discs. This extensive involvement made it challenging to differentiate from more common conditions, such as ossification of the CLF, ossification of PLL, or diffuse idiopathic skeletal hyperostosis (DISH) [16]. Accurate differentiation among these disorders is critical for appropriate diagnosis and treatment [12].

A definitive diagnosis of CPPD is established through pathological examination of affected tissues [17]. Once confirmed, treatment varies based on the presentation. Inflammatory responses may be managed conservatively with NSAIDs, colchicine, or glucocorticoids [18]. In cases where neural compression is significant, as in this patient, surgical decompression is required [14,19]. The preoperative imaging in our case demonstrated significant anterior compression by calcified PLL and intervertebral discs, as well as posterior compression from calcification in the CLF spanning multiple levels. A combined anterior and posterior approach was thus selected to remove compressive pathology from both sides and prevent residual or recurrent compression. In this patient, we performed a C3-C6 discectomy and a corpectomy at C4-C5 to thoroughly remove the calcified PLL, degenerated disc material, and any osteophytes contributing to anterior spinal cord compression. For the posterior compression, we performed a C2-C7 laminectomy to remove the calcified CLF. In terms of biomechanical stability, the anterior interbody fusion using a cage and anterior plate provided the main weight support. However, because of extensive laminectomy from C2 to C7, adding a posterior fusion was necessary to maintain stability, especially in the mid-cervical region most affected by pathology. By instrumenting C3-C6, we balanced the need for stabilization with a desire to preserve a degree of mobility above and below the fused levels. Careful surgical technique is necessary to avoid dural tears, as CPPD can strongly adhere to the dura [19]. Early surgical intervention is generally associated with improved long-term neurological outcomes [10,20].

Once CPPD is identified in the spine, clinicians should assess for other potential sites of crystal deposition, as CPPD can affect both spinal and peripheral joints. In this patient, persistent right shoulder pain after surgery was attributed to CPPD in the shoulder joint, highlighting the importance of a comprehensive approach to pain management in CPPD patients, which includes assessing for both nerve compression and peripheral joint involvement. This case illustrates the critical need to recognize CPPD as a potential cause of cervical myelopathy and serves as a valuable reference for its diagnosis and management.

## Conclusions

This case underscores that cervical CPPD can manifest with extensive ligament and disc involvement, leading to progressive, severe myelopathy that may mimic more common degenerative conditions or ossific processes. Prompt recognition is crucial for timely surgical decompression, which can alleviate neural compression and improve neurological outcomes. Furthermore, once histopathological evidence of calcium pyrophosphate crystals is confirmed, clinicians should remain vigilant for CPPD in peripheral joints, as multifocal crystal deposition may persist beyond the initial spinal presentation, emphasizing the need for comprehensive evaluation and management.
